# Malaria Parasite Invasion of the Mosquito Salivary Gland Requires Interaction between the *Plasmodium* TRAP and the *Anopheles* Saglin Proteins

**DOI:** 10.1371/journal.ppat.1000265

**Published:** 2009-01-16

**Authors:** Anil K. Ghosh, Martin Devenport, Deepa Jethwaney, Dario E. Kalume, Akhilesh Pandey, Vernon E. Anderson, Ali A. Sultan, Nirbhay Kumar, Marcelo Jacobs-Lorena

**Affiliations:** 1 Department of Molecular Microbiology and Immunology and Malaria Research Institute, Johns Hopkins School of Public Health, Baltimore, Maryland, United States of America; 2 Department of Immunology and Infectious Diseases, Harvard School of Public Health, Boston, Massachusetts, United States of America; 3 McKusick-Nathans Institute of Genetic Medicine and Departments of Biological Chemistry, Pathology, and Oncology, Johns Hopkins School of Medicine, Baltimore, Maryland, United States of America; 4 Department of Biochemistry, Case Western Reserve University, Cleveland, Ohio, United States of America; Stanford University, United States of America

## Abstract

SM1 is a twelve-amino-acid peptide that binds tightly to the *Anopheles* salivary gland and inhibits its invasion by *Plasmodium* sporozoites. By use of UV-crosslinking experiments between the peptide and its salivary gland target protein, we have identified the *Anopheles* salivary protein, saglin, as the receptor for SM1. Furthermore, by use of an anti-SM1 antibody, we have determined that the peptide is a mimotope of the *Plasmodium* sporozoite Thrombospondin Related Anonymous Protein (TRAP). TRAP binds to saglin with high specificity. Point mutations in TRAP's binding domain A abrogate binding, and binding is competed for by the SM1 peptide. Importantly, *in vivo* down-regulation of saglin expression results in strong inhibition of salivary gland invasion. Together, the results suggest that saglin/TRAP interaction is crucial for salivary gland invasion by *Plasmodium* sporozoites.

## Author Summary

Transmission of *Plasmodium*, the causative agent of malaria, requires the completion of a complex life cycle in the mosquito, which includes invasion of the salivary glands. This invasion depends on the recognition of mosquito salivary gland surface components by the parasite. This work demonstrates that interaction between the salivary-gland-specific surface protein saglin and the parasite surface protein TRAP is essential for invasion to occur. A better understanding of the mechanisms used by the parasite to develop in the mosquito may lead to novel approaches to intervene with the spread of the disease.

## Introduction

*Plasmodium* spp., the causative agent of malaria, is responsible for about 500 million clinical cases and about 2 million deaths every year, mostly of African children [1]. Furthermore, the number of cases is increasing [2] indicating that the available means to fight the disease are insufficient. Unlike the other two major infectious disease killers-AIDS and tuberculosis-malaria depends on an intermediate insect vector for transmission to occur. Thus, the complex life cycle of the parasite in the mosquito constitutes a potential weak link that could be exploited for malaria control.

*Plasmodium* transmission is initiated with the ingestion by the female mosquito, of gametocytes from an infected individual. Soon after ingestion, gametocytes differentiate into male and female gametes that mate to produce zygotes within the mosquito midgut. Within the next day, these differentiate into motile ookinetes that invade the midgut epithelium. After emerging on the hemocoel side, ookinetes differentiate into sessile oocysts that mature one or two weeks later (depending on *Plasmodium* species) releasing thousands of sporozoites into the open hemolymph circulation. Of all mosquito tissues and cell types that the sporozoites come in contact with, they invade only one: the salivary glands. The transmission cycle is completed when an infected mosquito bites another individual releasing some of its sporozoites.

Several lines of evidence suggest that sporozoite invasion of the salivary gland requires specific receptor-ligand interactions. For instance, *P. knowlesi* sporozoites can invade the salivary glands of *Anopheles dirus* but not salivary glands of *An*. *freeborni* [3]. Inhibition experiments with polyclonal serum against whole *Aedes aegypti* salivary glands and against specific salivary gland epitopes all suggest the involvement of specific receptors [4]–[6]. Moreover, a monoclonal antibody against the 100 kDa salivary gland protein inhibited invasion of *An. gambiae* salivary glands by *P. yoelii* sporozoites [7]. Recently it was shown that this monoclonal antibody recognizes a glycosylated homodimer protein made up of 50 kDa subunits, designated saglin [8]. Finally, binding of the SM1 peptide to *An. stephensi* salivary glands strongly interfered with *P. berghei* sporozoite invasion (see below).

Thrombospondin-related anonymous protein (TRAP) [9] is a protein expressed in sporozoites and conserved in all *Plasmodium* species. TRAP is essential for sporozoite gliding, cell invasion and *in vivo* infectivity [10]. TRAP is localized to the parasite micronemes [11] and becomes surface exposed at the sporozoite's anterior tip, particularly upon contact with the host [12]. It is also released onto the substrate during gliding locomotion [13]. The extracellular portion of TRAP contains two conserved adhesive domains: a von Willebrand factor A-domain (‘A-domain’) [14] and a thrombospondin type I repeat (TSR) [15]. This combination of TSR- and A-domains is also found in micronemal transmembrane proteins of many apicomplexans and independent lines of evidence suggest that these proteins have similar functions to TRAP [13], [16]–[19]. More recent results show that sporozoites carrying a mutated TRAP domain-A are impaired in salivary gland invasion but not in gliding motility suggesting that the two processes are functionally distinct and that the A-domain is involved in recognition and/or attachment to salivary gland receptor molecules [20],[21].

By use of a phage display library we had previously identified a peptide, SM1, which binds to the outer surface of anopheline salivary glands and inhibits sporozoite invasion, most likely because the peptide binds to and competes for a putative sporozoite receptor on the surface of the salivary glands [22]. This is true both for the rodent *P. berghei* parasite [22] and for the human *P. falciparum* parasite (unpublished observations) suggesting that salivary gland invasion of the two parasite species have similar pathways. However, the precise identity of the putative receptor and of the sporozoite ligand remained unknown. Here we show that SM1 is a mimotope of the TRAP domain-A and that SM1 interacts specifically with saglin on the surface of the salivary gland. Additional evidence suggests that TRAP domain-A directly interacts with saglin and that this interaction is essential for sporozoite invasion of the salivary gland.

## Results

### SM1 binds to a protein moiety on the salivary gland surface

**Figure 1:**
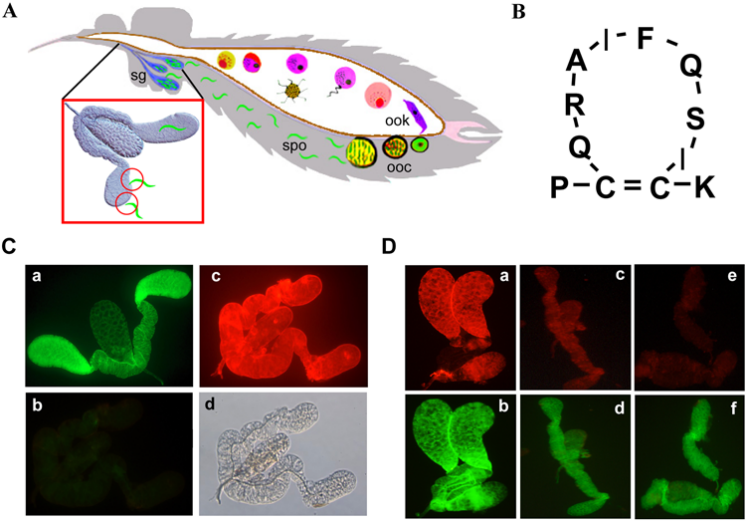
SM1 binds to a salivary gland protein. In all experiments the lectin (WGA) was labeled with FITC (green fluorescence) and binding of the biotinylated SM1 peptide was detected with Texas red-labeled streptavidin (red fluorescence). (A) *Schematic diagram of the lifecycle of malaria parasite in the mosquito vector*. After initial development in the midgut lumen, motile ookinetes (ook) cross the midgut epithelium and differentiate into sessile oocysts (ooc). Mature oocysts release sporozoites (spo) into the hemocoel and these specifically recognize the salivary glands (circled in the inset) resulting in invasion. (B) *SM1 structure*. The 12-amino acid SM1 peptide has two cysteine residues at positions 2 and 11 that when connected by disulphide bond form an 8-amino acid loop. (C) *Binding of SM1 to salivary glands does not involve surface carbohydrates*. Control salivary glands treated with buffer without periodate showed strong lectin binding (a). Panels (b–d) show an experimental gland treated with periodate and then incubated with a mixture of WGA and SM1. WGA bound poorly to this gland [b], indicating efficient removal of carbohydrates, while SM1 bound strongly (c), indicating that carbohydrate removal did not hinder SM1 binding. A differential interference contrast (DIC) image of the same gland is shown in (d). (D) *Protease treatment abrogates SM1 binding*. Salivary glands were treated with 0 (control; a, b), 25 µg/ml (c, d) or 100 µg/ml (e, f) of trypsin and were incubated with a mixture of SM1 and WGA. Protease treatment interfered with SM1 but not with lectin binding.

The SM1 peptide binds to two quite different epithelia, the midgut and the salivary gland, which are the two cell types that are invaded by *Plasmodium* parasites [22]. It was therefore possible that the SM1 peptide binds to a carbohydrate that is common to the two epithelia. In a preliminary test, we tested for the ability of eight different lectins to bind to salivary glands. Of these, WGA, jacalin, SBA and ConA bound strongly, DBA and PNA bound moderately and RCA and UEA bound weakly (Figure S1). When salivary glands were treated with sodium periodate, binding of WGA (Figure 1C) and several other lectins (results not shown) was lost, indicating that this treatment results in effective removal of surface carbohydrates. Importantly, binding of SM1 was unimpaired by periodate treatment (Figure 1C), suggesting that SM1 does not bind to a carbohydrate moiety. Treatment of salivary glands with three different glycosidases (PNGase and EndoH for N-linked carbohydrates, and *O*-glycosidase for O-linked carbohydrates) confirmed the results obtained with chemical carbohydrate removal (Figure S2A–C). Interestingly, while SM1 binds poorly to the proximal lobes [22] (Figure 1C), after carbohydrate removal SM1 effectively bound to this salivary gland region, indicating that carbohydrates may mask SM1 binding to the proximal lobes.

To test whether SM1 binds to a protein moiety, we repeated the assay with protease-treated salivary glands. Whereas protease treatment did not affect lectin binding, it severely inhibited SM1 binding (Figure 1D and Figure S2 D,E). These results suggest that SM1 binds to a surface protein.

### SM1 conformation mimics that of sporozoite TRAP domain-A

**Figure 2:**
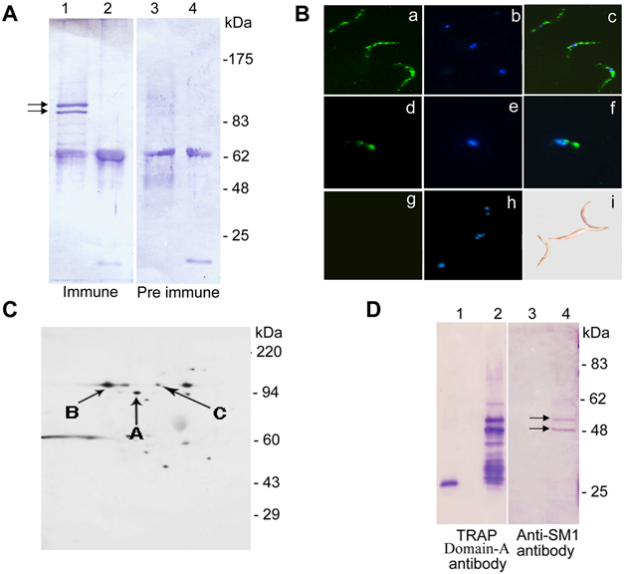
An anti-SM1 antibody recognizes a protein component of midgut sporozoites. (A) *Western blots with the anti-SM1 antibody*. The same number of midguts from *Plasmodium*-infected (carrying mature oocysts) or non-infected mosquitoes (mock) was used for gradient purification of sporozoites. Either 5×10^5^ sporozoites (lanes 1 and 3) or an equivalent amount of material from the mock purification (lanes 2 and 4) was fractionated by SDS-PAGE and blotted. One blot was incubated with anti-SM1 antibody (immune) and the other with pre-immune serum, as indicated. The double arrows point to the bands that are specifically detected by the immune serum. (B) *Indirect immunofluorescence staining (IFA)*. Fixed sporozoites were incubated with anti-SM1 or pre-immune serum followed by incubation with an FITC-labeled (green) secondary antibody. Nuclei were labeled with DAPI and appear blue. (a–c) Methanol-fixed (permeabilized) midgut sporozoites incubated with anti-SM1 antibody. (d–e) Paraformaldehyde-fixed (non-permeabilized) midgut sporozoites incubated with anti-SM1 antibody. (g–i) Methanol-fixed (permeabilized) midgut sporozoites incubated with pre-immune serum. Panel (i) is a DIC image of the same field as (g) and (h). (C) *Western blots of 2D gels with the anti-SM1 antibody*. Two aliquots of 10^6^ (samples 1 and 2) and one aliquot of 10^7^ (sample 3) sporozoites from the same enriched sporozoite preparation were fractionated in parallel by 2D gel electrophoresis. Gels from samples 1 and 2 were blotted onto a membrane and incubated with an anti-SM1 antibody (panel C) or with pre-immune serum (not shown). Sample 3 was stained with Coomassie Blue (not shown). Three protein spots (A–C) with mobility of ∼90 kDa (c.f. panel B) that reacted with the anti-SM1 antibody were excised from the stained gel for protein sequence analysis. (D) *Western blots with anti-TRAP and anti-SM1 antibodies.* Equal amounts of recombinant GST (lanes 1 and 3) and GST-tagged recombinant TRAP domain-A (lanes 2 and 4) proteins were fractionated by SDS-PAGE and the proteins blotted. The blot shown in the left panel was incubated with anti-TRAP domain-A antibody (the antibody was made against a GST-tagged recombinant protein) and the blot shown on the right panel was incubated with anti-SM1 antibody. The double arrows point to the TRAP domain-A bands recognized by the both antibodies.

Inhibition of sporozoite invasion of salivary glands by SM1 raised the possibility that the peptide shares sequence or structural similarity with a sporozoite protein involved in salivary gland recognition. Yet, exhaustive data base searches for *Plasmodium* proteins with sequence similarity to SM1 did not reveal any viable candidates. We reasoned that SM1 peptide conformation (rather than primary sequence) may be related to that of a sporozoite protein involved in salivary gland invasion. To test this hypothesis, we generated an anti-SM1 antibody. Immune (but not pre-immune) anti-SM1 serum specifically recognized an ∼95 kDa protein doublet on Western blots of purified sporozoites but not on blots of a mock-purified fraction from non-infected guts (Figure 2A). With immunofluorescence assays the anti-SM1 serum stained permeabilized (Figure 2B, a–c) with a patchy pattern. A small proportion (2∼3%) of the non-permeabilized sporozoites had a cap-like staining over one of its ends (Figure 2B, d–f). These staining patterns are typical of apicomplexan micronemal proteins that are released onto the parasite surface upon target cell contact, such as *Toxoplasma gondii* MIC2 [12],[16].

To identify the proteins that are recognized by the anti-SM1 antibody we performed 2D electrophoretic analysis with proteins from an enriched *P. berghei* sporozoite preparation (Figure 2C). Two aliquots of 10^6^ (samples 1 and 2) and one aliquot of 10^7^ (sample 3) sporozoites from the same purified sporozoite preparation were fractionated in parallel by 2D gel electrophoresis. Gels from samples 1 and 2 were blotted onto membranes and incubated with an anti-SM1 antibody (Figure 2C) or with pre-immune serum (not shown). Sample 3 was stained with Coomassie Blue (not shown). Three major stained protein spots (A–C) with mobility of ∼90 kDa that reacted with the anti-SM1 antibody were excised from the stained gel and sequenced by mass spectrometry. Protein B (Figure 2C) corresponded to the secreted *P. berghei* TRAP protein while the other two corresponded to the *Plasmodium* cytoplasmic hsp90 protein. SM1 and the domain-A of *P. berghei* TRAP share some amino acid sequence similarity (Figure S3). We produced GST-tagged recombinant TRAP domain-A (and GST as a control) and determined that TRAP domain-A antibodies and anti-SM1 antibodies recognize proteins of similar mobility (Figure 2D). Together, these results indicate that SM1 is a conformational analog of TRAP domain-A. Additional evidence supporting this hypothesis is presented below.

### Pull-down assays reveal that SM1 binds to the salivary gland surface protein saglin

**Figure 3:**
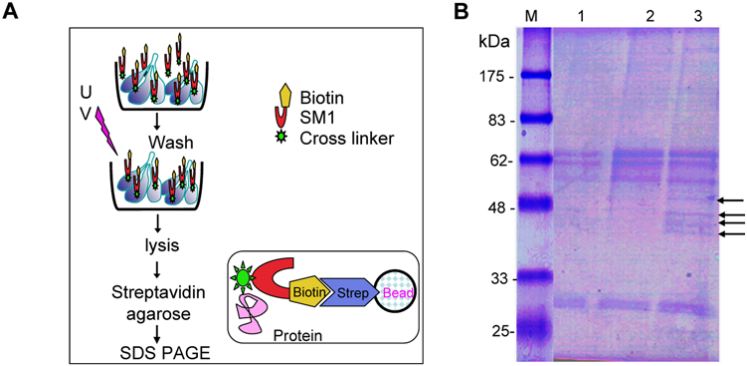
Pull-down of salivary gland proteins that interact with SM1. (A) *Schematic diagram of the pull-down approach*. A double-derivatized SM1 peptide, with a biotin residue (yellow pentagon) at the N-terminus and a UV-crosslinking residue (green star) attached to a phenylalanine residue in the loop (‘F’ in Figure 1B) was incubated *in vitro* with freshly dissected salivary glands and washed. After shining UV to promote crosslinking of the peptide to the protein to which it was bound, the salivary glands were lysed and the peptide with the crosslinked protein was captured on streptavidin beads. The beads were washed and the retained proteins were fractionated by SDS-PAGE (panel B). (B) *Gel electrophoresis of proteins captured on the streptavidin beads*. Materials recovered from a pull-down experiment illustrated in panel A were fractionated by SDS-PAGE under reducing conditions and the gel was stained with Coomassie Blue. Lane 1, Materials eluted from streptavidin beads that were not incubated with any proteins. The stained bands are presumed to be bead-derived contaminants. Lane 2, Complete experiment, except that the crosslinking step was omitted. Lane 3, complete experiment, including the crosslinking step (same number of salivary glands as in Lane 2). The four arrows point to protein bands consistently observed only in the complete experiment.

To identify salivary gland protein(s) to which SM1 bound, we used a double-derivatized SM1 peptide that had biotin at the N-terminus and an UV-crosslinker attached to the phenylalanine (F) within the 8-amino acid loop formed by the disulphide bond between cysteines 2 and 11 (Figures 1B and 3A). The derivatized SM1 was incubated with freshly dissected salivary glands followed by UV irradiation to activate peptide crosslinking to its target salivary gland receptor (Figure 3A). The glands were then solubilized and the peptide, with its crosslinked proteins, was captured on a streptavidin column. The retained proteins were then fractionated by gel electrophoresis. The four specific protein bands (Figure 3B) were excised and microsequenced. The upper two bands corresponded to the recently described mosquito salivary gland surface protein saglin [8] whereas the two lower bands corresponded to the mosquito salivary gland secreted protein SG1 of unknown function. Figure S3 illustrates the identification of saglin by LCMS/MS. Saglin has a predicted secretion signal sequence at its amino terminus and is rich in the amino acid glutamine (47/412 amino acids or 11.4%) that may be involved in protein-protein interactions via hydrogen bonds. Monoclonal antibodies recognizing saglin had previously been shown to inhibit sporozoite invasion of salivary glands [7].

### *In vitro* interactions of saglin with TRAP domain-A and SM1

**Figure 4:**
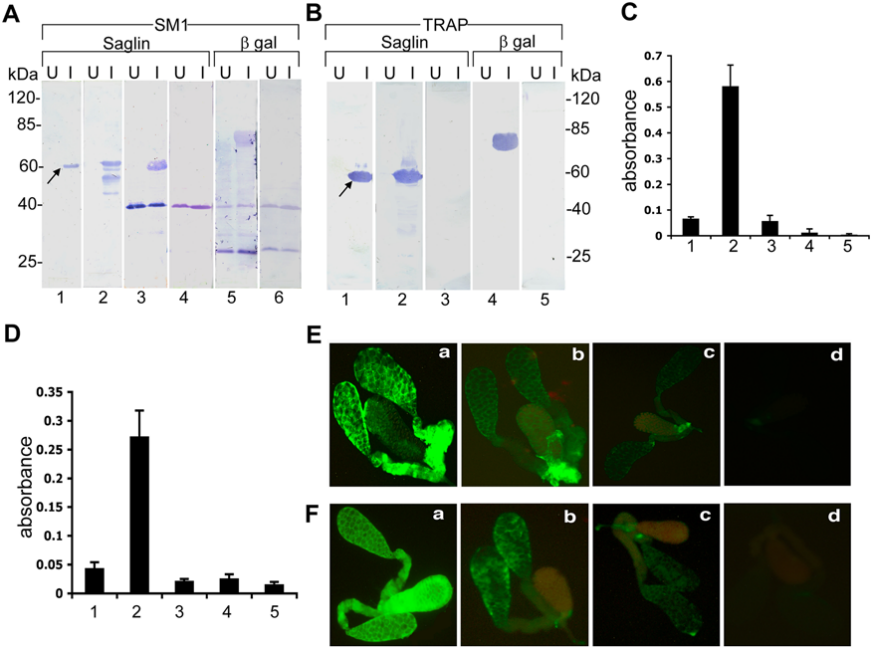
*In vitro* and *in vivo* protein interactions. (A) *SM1 binding to saglin on protein blots*. Blots from bacteria carrying a plasmid encoding either histidine-tagged saglin (experimental; panels 1–4) or histidine-tagged ß-galactosidase (control; panels 5–6) were produced from proteins recovered either before (U) or after (I) induction of recombinant protein synthesis. Panel 1: Experimental blot incubated with anti-histidine antibody. Panel 2: Experimental blot incubated with anti-saglin monoclonal antibody. Panel 3: Experimental blot first incubated with the SM1 peptide (10 µg/ml) and then probed with the anti-SM1 antibody followed by incubation with an alkaline phosphatase-tagged secondary antibody. Panel 4: Experimental blot incubated with a linear SM1 peptide and probed with anti-SM1 antibody. In the linear peptide the two cysteines were substituted by alanines. In separate experiments the linear peptide was unable to bind to salivary glands (not shown). Panel 5: Control blot probed with anti-histidine antibody. Panel 6: Control blot first incubated with the SM1 peptide (10 µg/ml) and then probed with the anti-SM1 antibody. The mobility of recombinant saglin is indicated by an arrow in Panel 1. (B) *TRAP domain-A binding to saglin on protein blots*. Blots from bacteria carrying a plasmid encoding either histidine-tagged saglin (experimental; panels 1–3) or histidine-tagged ß-galactosidase (control; panels 4–5) were produced from proteins recovered either before (U) or after (I) induction of recombinant protein synthesis. Panel 1: Experimental blot incubated with anti-histidine antibody; Panel 2: Experimental blot first incubated with recombinant TRAP domain-A protein (2.5 µg/ml) and then probed with anti-TRAP domain-A antibody, followed by incubation with an alkaline phosphatase-tagged secondary antibody; Panel 3: Same as Panel 2, except that incubation with anti-TRAP domain-A antibody was omitted; Panel 4: Control blot probed with anti-histidine antibody; Panel 5: Control blot subjected to the same treatment as described for Panel 2. The mobility of recombinant saglin is indicated by an arrow in panel 1. (C) *In vitro interaction between saglin and TRAP domain-A.* Recombinant TRAP domain-A was incubated in wells of an ELISA plate that had been previously coated with recombinant histidine-tagged saglin. The wells were washed and TRAP binding was quantified using a TRAP antibody followed by incubation with an alkaline phosphatase-tagged secondary antibody. Results of the colorimetric alkaline phosphatase reaction are reported. (1) Well not coated with recombinant saglin. (2) Complete protocol. (3) Incubation with TRAP omitted. (4) Incubation with anti-TRAP antibody omitted. (5) Incubation with secondary antibody omitted. (D) *Sporozoites bind to saglin in vitro*. Purified midgut sporozoites were incubated in wells of an ELISA plate that had been previously coated with recombinant histidine-tagged saglin. The wells were washed and sporozoite binding was quantified by use of an anti-CS antibody followed by incubation with an alkaline phosphatase-tagged secondary antibody. Results of the colorimetric alkaline phosphatase reaction are reported. (1) Well not coated with recombinant saglin. (2) Complete protocol. (3) Incubation with sporozoites omitted. (4) Incubation with anti-CS antibody omitted. (5) Incubation with secondary antibody omitted. (E) *SM1 competes with TRAP domain-A for binding to salivary glands*. Fixed salivary glands were first incubated with no (a), 0.5 (b), 5 µM (c) or 50 µM (d) of non-biotinylated SM1 peptide followed by incubation with 50 nM of recombinant TRAP domain-A. TRAP domain-A binding was detected by incubation with an anti-TRAP antibody followed by incubation with a FITC-labeled secondary antibody. Note that the SM1 peptide effectively competes with TRAP domain-A for binding to salivary glands. (F) *Reverse competition: TRAP domain-A competes with SM1 for binding to salivary glands*. Fixed salivary glands were first incubated with no (a), 20 nM (b), 200 nM (c) or 2 µM (d) of recombinant TRAP domain-A protein followed by incubation with biotinylated SM1 peptide (5 µM). Peptide binding was detected by incubation with FITC-labeled streptavidin. Note that the protein effectively competes with the SM1 peptide for binding to salivary glands.

The results presented so far suggested that SM1 mimics the conformation of TRAP domain-A and that SM1 interacts with saglin. Further experiments were conducted to verify that these components interact directly. Figure 4A and 4B present results of far-western experiments in which bacterial lysates containing either recombinant saglin or control beta-galactosidase were fractionated by gel electrophoresis and blotted. The blot in Figure 4A was incubated with the SM1 peptide and the blot in Figure 4B was incubated with TRAP domain-A and peptide or TRAP domain-A binding was detected with their respective antibodies. Panel 3 of Figure 4A shows that SM1 binds to saglin and panel 2 of Figure 4B shows that TRAP domain-A binds to saglin. Binding was specific and was not detected when the control beta-galactosidase was run on the gel (Figure 4A panel 6 and Figure 4B panel 5).

Additional evidence was obtained for the direct interaction between saglin and TRAP domain-A in the following experiment. Recombinant saglin was captured on wells of a microtiter plate followed by incubation with recombinant TRAP domain-A. As seen in Figure 4C, interaction between saglin and TRAP domain-A was detected only in the complete experiment (bar 2) and not when one of the steps was omitted, attesting to the specificity of the interaction. These results confirmed that TRAP domain-A directly interacts with saglin. In separate experiments, we trapped salivary gland membranes (and ovary membranes as a control) on wells of a microtiter plate that had been pre-coated with anti-saglin antibodies. TRAP domain-A bound to salivary gland but not to ovary membranes (Figure S5), again suggesting tissue specificity of interaction.

### Midgut sporozoites bind to saglin

**Figure 5:**
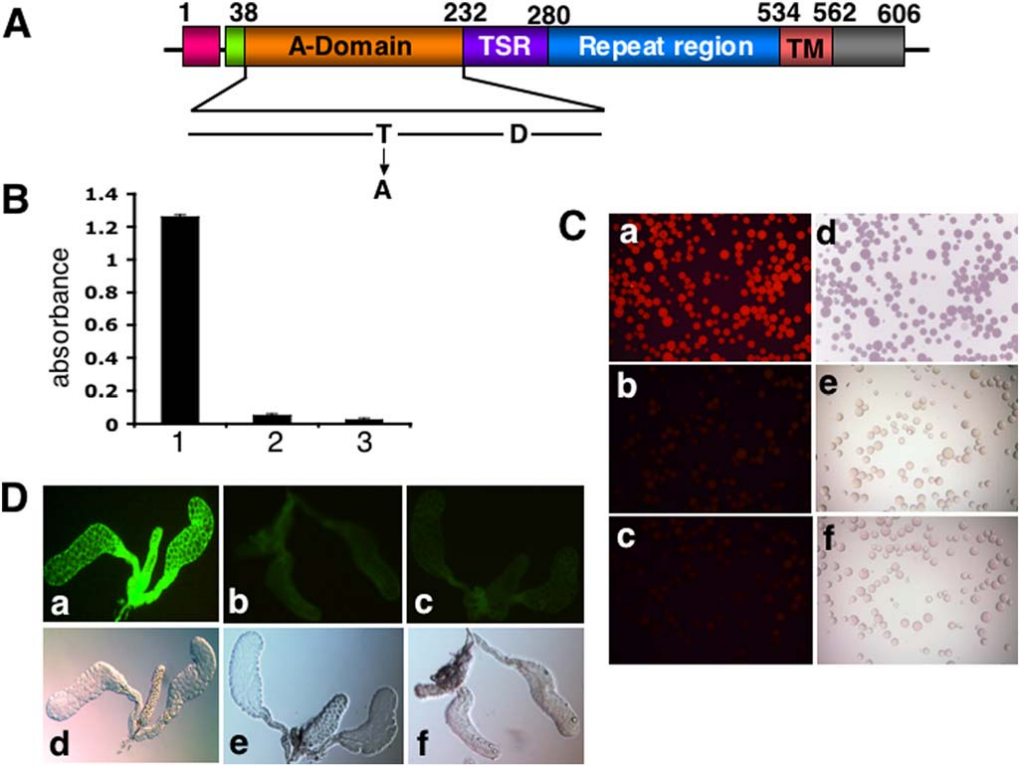
Mutational analysis of TRAP-saglin interactions. (A) Diagram of the TRAP protein and the Thr_126_ to Ala mutation in the MIDAS domain. (B) Binding of recombinant wild type or mutant TRAP domain-A to saglin. Saglin was captured on wells of a nickel-coated plate and wild type or mutant recombinant TRAP domain-A were added to the wells, incubated and washed. Bound TRAP domain-A was detected by incubation with anti-TRAP domain-A antibody followed by an alkaline phosphatase-conjugated secondary antibody. (1) Wild type TRAP domain-A; (2) Point mutant TRAP domain-A; (3) No TRAP protein added. (C) Direct visualization of the binding of wild type and mutant TRAP domain-A protein to recombinant saglin. Histidine-tagged saglin was immobilized on Ni-agarose beads, washed and allowed to bind to the purified wild type or mutant TRAP domain-A recombinant protein. Beads were washed and incubated with anti-domain-A antibody. Detection was done after incubation with Alexa Flour-568-conjugated anti-rabbit IgG (red). (a) Wild type TRAP domain-A; (b) Point mutant TRAP domain-A; (c) No TRAP protein added. Panels (d), (e) and (f) show light microscopic images of the fields to their left. (D) Binding of recombinant wild type and mutant domain-A to salivary glands. Fixed salivary glands were incubated with wild type or mutant TRAP domain-A protein, washed and incubated with anti-TRAP domain-A antibody. Detection was with anti-rabbit IgG conjugated to Alexa Flour-488 (green). (a) Wild type TRAP domain-A; (b) Point mutant TRAP domain-A; (c) No TRAP protein added. The lower panels (d, e, f) show light microscopic images of the fields on top (a, b, c, respectively).

The preceding results raised the possibility that for salivary gland invasion, sporozoites anchor to saglin on the surface of salivary glands via its TRAP protein. To provide further evidence for this hypothesis we incubated purified sporozoites in microtiter plate wells previously coated with recombinant saglin. The results show that sporozoites bind to the wells only when they are coated with saglin (Figure 5D), suggesting a direct interaction between sporozoites and saglin.

### TRAP domain-A binds to salivary glands and the SM1 peptide competes for this binding

The findings that the SM1 peptide ‘pulls-down’ saglin from the salivary gland surface and that anti-SM1 antibodies recognize TRAP on the sporozoites surface suggested that sporozoite TRAP protein mediates salivary glands invasion by binding to one of its components. We tested this prediction by incubating recombinant TRAP domain-A with salivary glands and found that indeed the protein binds directly to its surface (Figure 4E, panel a). We also investigated whether this interaction could be competed away with the SM1 peptide. As seen in Figure 4E (panels a–d), SM1 competed in a dose dependent manner with TRAP domain-A for binding to salivary glands. At 0.5 µM only partial inhibition was observed while higher concentrations effectively prevented TRAP domain-A binding. In a complementary set of experiments, we demonstrated that binding of TRAP domain-A to the salivary gland surface effectively prevents SM1 binding (Figure 4F). These results indicated that the SM1 peptide and TRAP domain-A compete for the same receptor on the salivary gland surface and this is most likely saglin.

### Mutational analysis of TRAP-saglin interactions

TRAP domain-A contains a MIDAS (metal-ion-dependent adhesion site) motif that is essential for sporozoite invasion of salivary glands [10]. In particular, when the critical Thr_126_ amino acid is mutated to an alanine, sporozoite invasion of mosquito salivary glands is strongly (∼80%) impaired [21]. However, the molecular basis for this impairment is not understood. We have produced a recombinant TRAP domain-A protein carrying the Thr_126_-to-alanine mutation (Figure 5A) and shown that the mutation abrogates the ability of the protein to bind to saglin (Figure 5B and 5C). In addition, binding of the mutant protein to mosquito salivary glands was also abrogated (Figure 5D). These results attest to the specificity of the interaction between TRAP domain-A and mosquito salivary glands or recombinant saglin.

### Saglin is required for sporozoite invasion of salivary glands

**Figure 6:**
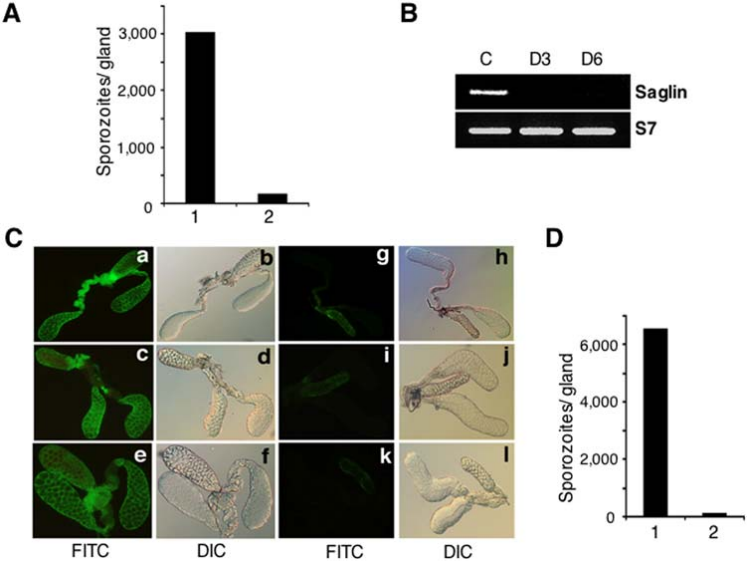
Sporozoites interact with saglin and this interaction is essential for salivary gland invasion *in vivo*. (A) Inhibition of *P. falciparum* sporozoite invasion of *An. gambiae* salivary glands by anti-saglin antibody. Either anti-saglin monoclonal antibody [1.5∼2 µg, bar 2] or an equivalent amount of an unrelated antibody [control; bar 1] was injected into the haemocoel of 50 *P. falciparum*-infected *An. gambiae* female mosquitoes. At 48 h after injection the salivary glands of 22 to 30 surviving mosquitoes were dissected and the number of sporozoites determined. A 95% inhibition was observed in this experiment (*p*-value, <0.0001). The inhibition in a second experiment (not shown) was 94% (*p*-value, <0.0001). (B) *Down-regulation of saglin mRNA abundance by RNA interference*. Either control double-stranded GFP RNA (C) or double-stranded saglin RNA was injected into the hemocoel of *An. gambiae* female mosquitoes and salivary glands were dissected on days 3 (D3) and 6 (D6) after injection, as indicated. Saglin mRNA abundance was assessed by semi-quantitative PCR using ribosomal protein S7 mRNA primers as controls. (C) *Down-regulation of saglin protein abundance by RNA interference*. Double-stranded GFP RNA (control; panels a–f) or double-stranded saglin RNA (experimental, panels g–l) was injected into the hemocoel of female *An. gambiae* mosquitoes and salivary glands were dissected after 1 d (a, b, g, h), 3 d (c, d, i, j) or 6 d (e, f, k, l) for immunofluorescence assays (IFA) with an anti-saglin antibody. The panels pair fluorescent images to the left and DIC images of the same gland to the right. (D) *Inhibition of sporozoite salivary gland invasion after down-regulation of saglin expression*. Approximately 50 *P. falciparum*-infected *An. gambiae* mosquitoes were injected with GFP (1) or saglin (2) dsRNA. Salivary glands were dissected from each of 15∼18 surviving mosquitoes and the number of sporozoites determined. Inhibition was 89% (*p*-value <0.0008). The inhibition in a second experiment (not shown) was 97% (*p*-value <0.0001).

To evaluate the importance of saglin for *P. falciparum* sporozoite invasion of salivary glands, we injected mouse anti-saglin monoclonal antibody into infected *An. gambiae* mosquitoes at a time when sporozoites are starting to invade the salivary glands. Two days later, we assayed the number of sporozoites that had invaded the salivary glands. As controls, mosquitoes from the same cohort were injected with an unrelated monoclonal antibody. We found that the antibody strongly inhibited *P. falciparum* sporozoite salivary gland invasion (Figure 6A), suggesting that saglin plays a central role in this process. Similar results were obtained for experiments with *P. berghei* sporozoite invasion (results not shown). Previous work had shown that the same monoclonal antibody inhibits salivary gland invasion by the rodent *P. yoelii* parasite [7]. A possible caveat for the interpretation of these results is that saglin may not be directly recognized by the sporozoites but rather, that inhibition is due to steric hindrance by the bulky antibody. To address this possibility, we used RNA interference to knockdown saglin expression. Injection of saglin double-stranded (ds) RNA led to strong decrease of its mRNA abundance and this downregulation was maintained for at least 6 d (Figure 6B). We also used immunofluorescence assays to follow the abundance of saglin protein on the salivary gland surface after saglin dsRNA injection and found that there was a strong decline in protein abundance in saglin dsRNA-injected mosquitoes but not in GFP dsRNA-injected control mosquitoes (Figure 6C). Finally, we determined the effect of saglin depletion on the ability of sporozoites to invade the salivary gland. Saglin dsRNA, or GFP dsRNA as a control, were injected into mosquitoes infected with *P. falciparum* and the number of sporozoites that had invaded the salivary gland was assayed 6 d later. Sporozoite invasion of salivary glands in mosquitoes injected with saglin dsRNA was inhibited by 89 to 98% relative to GFP dsRNA-injected mosquitoes (Figure 6D). These results show that saglin plays a crucial role in sporozoite invasion of salivary glands.

## Discussion

**Figure 7:**
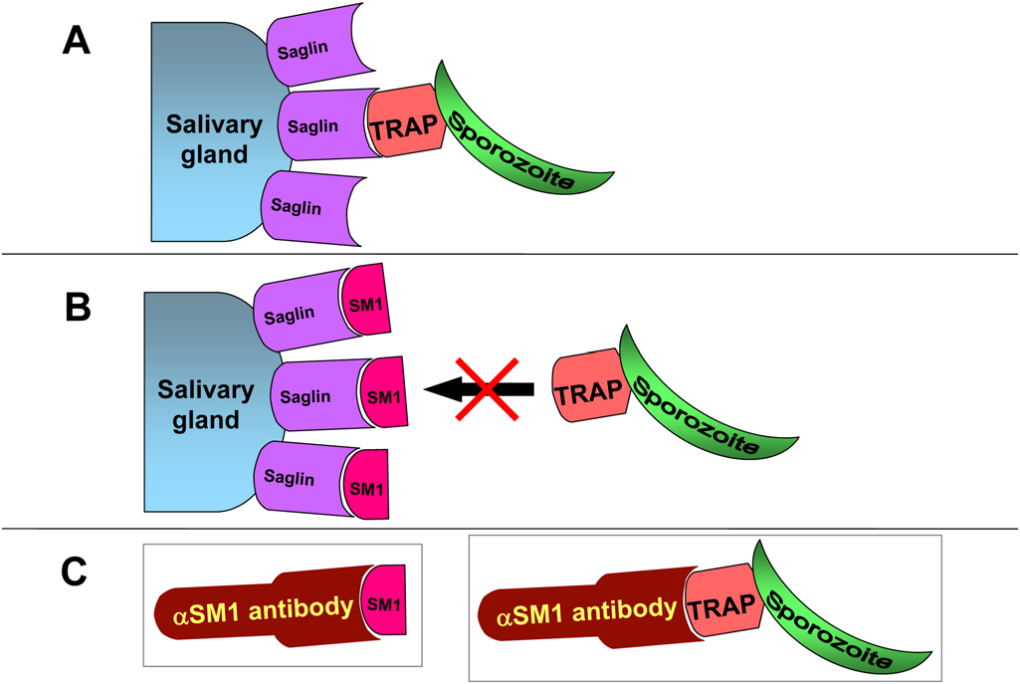
Schematic diagram of the presumed interaction among the various proteins used in this study. (A) Invasion of salivary glands requires the interaction between the *Anopheles* salivary gland surface protein saglin with the *Plasmodium* secreted protein TRAP. (B) The SM1peptide also interacts with saglin because its conformation mimics that of TRAP. In the presence of SM1 all saglin sites are occupied, thus preventing saglin-TRAP interactions and interfering with sporozoite invasion. (C) Because the conformations of SM1 and TRAP are related, an anti-SM1 antibody also recognizes TRAP. This relationship led to the identification of TRAP.

Salivary gland invasion is an essential step of the *Plasmodium* life cycle in its mosquito vector. Circumstantial evidence suggests that this invasion process is mediated by specific receptor-ligand interactions. Here we report that the salivary gland protein saglin is a receptor and that the sporozoite protein TRAP is a ligand in this invasion. Both proteins had independently been implicated in the process of invasion [7],[20],[21]. Saglin is expressed specifically on the salivary gland distal lobes, which is the region invaded by sporozoites [7]. Coincidently, the SM1 dodecapeptide (PCQRAIFQSICN) that was originally isolated from a phage display library screen for phages that bind to the surface of salivary glands also binds specifically to the salivary gland distal lobes. Moreover, inhibition of sporozoite invasion by SM1 binding to the salivary glands [22] raised the possibility that SM1 competes with a sporozoite protein for a receptor on the salivary gland surface (Figure 7). Here we report that SM1 binds to saglin on the mosquito salivary gland and that an anti-SM1 antibody recognizes the sporozoite protein TRAP (Figure 7). These findings led to the concept that the two proteins may interact, a hypothesis that was verified by further *in vitro* and *in vivo* experiments. Our results indicate that both *P. falciparum* and *P. berghei* use saglin as a receptor. The fact that the SM1 peptide blocks salivary gland invasion of both *P. berghei* [22] and *P. falciparum* (unpublished results) makes it likely that both parasite species use TRAP domain-A as a ligand for invasion.

As a search of available databases did not reveal any *Plasmodium* protein with similarity to the peptide, we reasoned that SM1 conformation, rather than primary amino acid sequence, is related to a sporozoite protein. Several observations supported this assumption. First, SM1 binding to salivary glands was abolished by the reduction of the disulphide bond between the two cysteines (unpublished observation). Second, an anti-SM1 antibody recognizes sporozoite TRAP (Figure 2A and 2D). Third, TRAP domain-A binds to salivary glands and this binding can be competed by the SM1 peptide (Figure 4E and 4F). TRAP-related proteins mediate cell-cell and cell-matrix interactions in a wide variety of biological systems [23]. Moreover, TRAP is an essential protein for gliding motility and sporozoite invasion of mosquito salivary glands [10]. Sporozoites harboring mutations in TRAP domain-A (the part of the protein that resembles SM1) preserve gliding motility but are impaired in salivary gland invasion [21]. Like TRAP, adhesive domains of CTRP and WARP also mediate ookinete invasion of the mosquito midgut epithelial cells [18], [24]–[26]. TRAP domain-A harbors an important module termed metal ion-dependent adhesion site (MIDAS). Within this module threonine (T_126_) and aspartate (D_157_) are conserved in all *Plasmodium* species. In a vertebrate model system, mutation of these residues to alanines prevent divalent cation ( Mg^2+^ or Mn^2+^) coordination and A-domain binding to its ligand, without affecting A-domain folding [27]. We showed that the T126-to-Ala mutation abrogates binding to salivary glands or to saglin *in vitro*. These results suggest that TRAP domain-A interaction with salivary glands is mediated by binding to saglin. Alignment of SM1 to the amino acid sequence of domain-A revealed that 6 out of 8 amino acids in the SM1 loop between the two cysteines loosely aligned within a region of the MIDAS domain (Figure S4).

We used a benzophenone photophore to crosslink SM1 to the salivary gland protein(s) with which it interacts. This photo-crosslinker has several advantages for the study of peptide-protein interactions: (a) it is chemically more stable than other photoactivatable reagents; (b) it reacts preferentially with C–H bonds even in the presence of water; (c) it is compatible with peptide synthesis methodology; and d) it has a broad 300–600 nm action spectrum [28],[29]. Initial experiments with a SM1 peptide carrying the benzophenone moiety on the C-terminal end were not successful but changing its position to the phenylalanine in the SM1 loop led to the identification of saglin. We presume that this is because the SM1 loop fits into a receptor pocket, bringing the crosslinking agent into close proximity of receptor amino acids [30].

SM1 peptide pull-down assays identified two candidate salivary gland proteins, saglin and an SG1-like protein. Saglin and the SG1-like candidate protein share only 20% sequence similarity. SG1 belongs to a family encoding a complex of high molecular weight proteins among which is a candidate receptor [6]. The possible role of the SG1 candidate protein identified in this study in sporozoite invasion of salivary glands remains to be determined. Saglin was previously identified from the analysis of a monoclonal antibody against salivary glands that effectively inhibits *P. yoelii* sporozoite invasion of *An. gambiae* salivary glands [7]. The following evidence supports the concept that saglin is present on the outer surface of the salivary glands, facing the hemocoel. 1) Immuno-electronmicrographs by Brennan et al. [7] established that saglin is present at this location. 2) Injection of anti-saglin antibody (but not of a control antibody) into the hemocoel of living mosquitoes strongly inhibits sporozoite invasion (Figure 6A). These results are only compatible with the presence of saglin on the salivary gland surface facing the hemocoel. The saglin gene is predicted to encode a 50 kDa glycoprotein harboring a signal sequence at the N-terminus [8]. However, no transmembrane domain or GPI consensus domain could be identified in the deduced amino acid sequence of the protein. There is precedent for surface proteins without transmembrane domains, such as enolase [31] and the IL2 receptor [32]. However, the mechanisms of surface attachment by such proteins are not well understood. Glutamines can promote protein-protein interactions through hydrogen bond formation [33] and it is possible that the relative high abundance of glutamine residues in saglin perform this function. The resolution of previously published immunoelectronmicrographs [7] is not sufficient to determine whether saglin is part of the salivary gland surface or of the basal lamina.

There is evidence that other sporozoite proteins also play a role in salivary gland invasion. The synthetic peptide (KLKQP) from the circumsporozoite (CS) protein region I inhibits *P. berghei* sporozoite salivary gland invasion [34] but the receptor of this peptide has not been identified. Parasites harboring a deletion of CS region II lost their gliding motility and infectivity of the salivary gland [35] but how region II interacts with the salivary gland surface is unknown. Another micronemal protein, MABEL, is also expressed on the sporozoite surface. MABEL knockout parasites showed a reduction in the salivary gland infectivity [36] but no salivary gland MABEL receptor has been identified.

This work provides compelling evidence for a central role that the interaction between *Anopheles* saglin and *Plasmodium* TRAP plays in sporozoite invasion of the salivary gland. It should be emphasized however that sporozoite invasion of the salivary gland is a complex process [37] and that the interactions identified in this study represent only one of many necessary steps. Whereas the road to a complete understanding of the molecular basis of invasion is likely to be long, elucidation of these mechanisms may lead to novel approaches to intervene with the spread of malaria.

## Materials and Methods

### Mosquitoes

Colonies of *An. gambiae* (G-3 strain) and *An.stephensi* (Dutch strain) were established from colonies maintained at the Laboratory of Parasitic Diseases (National Institute of Allergy and Infectious Diseases, National Institute of Health, Bethesda). Larvae were reared on dry cat food. Adults were maintained on 10% sucrose solution at 27±1°C and 80±5% relative humidity with a 14h/10h light/dark cycle.

### Parasites

*P. falciparum* strain NF54 gametocyte cultures were maintained as described [38]. Gametocyte-rich cultures were fed to female *An. gambiae* using a glass membrane feeder. *P. berghei* strain ANKA 2.34 was maintained by passage in Swiss Webster mice [39],[40].

### Lectins

The following FITC-labeled lectins were purchased from Vector Laboratories (California]: wheat germ agglutinin (WGA), jacalin, soybean agglutinin (SBA), concanavalin A (ConA), *Dolichos biflorus* agglutinin (DBA), peanut agglutinin (PNA), *Ricinus communis* agglutinin (RCA) and *Ulex europaeus* agglutinin (UEA).

### Derivatized peptides and anti-peptide antibody

The biotinylated SM1 peptide was synthesized by Genemed Synthesis (South San Francisco, California). For immunizations, the asparagine residue of the original SM1 peptide sequence was changed to lysine (K) [PQRAIFQSICN/K] for conjugation to Keyhole Limpet Hemocyanin (KLH) using the glutaraldehyde method [41]. The final conjugate was purified by gel filtration and used to immunize rabbits (Biosynthesis Inc., Lewisville, Texas, USA). A double-derivatized (photocrosslinker and biotin) SM1 peptide was synthesized by Biosynthesis Inc. using a Bpa (*p*-benzoyl -L- phenylalanine) photocrosslinker residue that was incorporated into the SM1 peptide at the phenylalanine residue [29]. After synthesis the peptide was cyclized at the cysteine residues and biotin was added at the N-terminal end. Binding activity of the biotinlyated circular peptide to mosquito salivary glands was verified by immunofluorescence assays [22].

### Chemical carbohydrate removal from salivary glands

Salivary glands were dissected and incubated in periodate buffer (50 mM sodium acetate, 100 mM NaCl, pH 5.5) for 1 h at room temperature before treatment [42], and then transferred to periodate solution (50–100 mM) for 12 h at 4°C in the dark. After treatment, glands were thoroughly washed in PBS and fixed in 4% paraformaldehyde overnight at 4°C. Removal of surface sugars was confirmed on sample salivary glands using eight different FITC-labeled lectins: WGA, SBA, ConA, jacalin, RCA, PNA, UEA, and DBA.

### Enzymatic carbohydrate removal from salivary glands

Salivary glands were treated with 20 mU PNGase F (Prozyme, California), 5 mU Endo H or 5 mU *O*-glycosidase (Roche applied Science, Annapolis USA) in the following buffers: 100 mM sodium phosphate with 0.1% sodium azide for PNGase; 50 mM sodium acetate pH 6.0 for Endo H; and phosphate buffer pH 7.4 for *O*-glycosidase. All enzyme treatments were done overnight at room temperature in 50 µl final volume and in the presence of a protease inhibitor cocktail. After enzymatic digestion, salivary glands were washed several times in PBS buffer and fixed in 4% paraformaldehyde overnight at 4°C. Removal of surface sugars was confirmed on sample salivary glands using the FITC-conjugated lectins jacalin (for N-linked glycosidase) and WGA (for O-linked glycosidase).

### Purification of midgut sporozoites

Midgut sporozoites were purified by a method modified from Barreau et al. [4]. *An. stephensi* mosquitoes were fed on *P. berghei* infected mice (3–5% parasitemia, 0.2–0.5% gametocytemia, and exflagellation rate 1 to 2 per field at 40× magnification). Fed mosquitoes were collected and kept at 19°C with 10% sucrose. On day 15, mosquito midguts were dissected and loosely homogenized with a plastic pestle in PBS, and cell debris were removed by passing through a glass wool column. Sporozoites that passed through the column were concentrated by gentle centrifugation and washed several times in PBS. The final pellet was layered on a cushion of 6.4% Ficoll (Pharmacia, Texas) and 10% Hypaque (Sigma) and centrifuged at 4,000 *g* for 15 min at room temperature. A fraction±5 mm from the cushion interface was recovered and the sporozoites were washed in PBS. Sporozoite numbers were determined using a hemocytometer.

### 2D gel electrophoresis and Western blot analysis

Proteins from Ficoll-Hypaque-purified midgut *P. berghei* sporozoites were separated by two-dimensional gel electrophoresis AS DESCRIBED [43],[44]. Three identical gels were run in parallel, two for blotting and a third to recover proteins for sequencing by tandem mass spectrometry. One of the blots was incubated with the anti-SM1 antibody (1:1000 dilution), and another one with preimmune serum for 1 h at room temperature, washed and then incubated with alkaline phosphatase-conjugated anti-rabbit IgG secondary antibody. The third gel was stained with Coomassie Blue. Stained spots corresponding to the anti-SM1 positive proteins were excised, washed and digested with trypsin prior to LC-MS/MS analysis as described below in the Mass Spectrometry (MS) analysis section.

### Far-Western blotting

Bacteria carrying protein expression plasmids were induced or not with arabinose (0.2%). After induction, cells were grown for another 4 h at room temperature and an aliquot of each culture (equal number of cells) was centrifuged. The final pellet was resuspended in 20 µl of sample buffer, and lysed by boiling. The samples were centrifuged again and the soluble proteins were fractionated by electrophoresis in a 10% SDS-PAGE before transferring onto PVDF membrane (Millipore). After transfer, membranes were washed with several changes in TBST buffer (150 mM NaCl, 20 mM Tris-HCl,10 mM MgCl_2_ and 0.1% Tween-20, pH 7.5), blocked in TBST with 4% BSA, and incubated with biotinylated SM1 peptide (10 µg/ml) or recombinant TRAP domain A (2.5 µg/ml) in blocking buffer. After incubation, blots were washed in TBST and incubated either with alkaline phosphatase(AP)-labeled streptavidin (peptide blots) or with anti-TRAP domain-A antibody (1:1000,dilution) in blocking buffer for 1 h followed by incubation with AP-conjugated anti-rabbit IgG (1:5000 dilution, Promega). Blots were then washed in TBST several times and 3 times with TBS followed by incubation with the substrate solution (NBT,BCI P, solution, Western Blue stabilized substrate for Alkaline Phosphatase (Promega). After visualizing the bands, color reaction was stopped with stop solution (1 mM EDTA, 10 mM Tris-HCl, pH 8.0). The final blot was air dry and pictures were taken.

### Peptide pull-down assays

Salivary glands were dissected and kept ice-cold in the presence of protease inhibitors before washing thoroughly with several changes of PBS to remove cell debris and other contaminating materials. The glands were then incubated in a shallow chamber with the double-derivatized SM1 peptide in PBS (10 µg/ml) for 1 h at 4°C in the dark. Glands were washed to remove unbound peptide, transferred to another ice-cold shallow chamber, and exposed to UV light for 30 min using a hand-held UV lamp (240 nm wave length) held at a distance of 1 cm from the glands. The glands were then transferred to a microcentrifuge tube, centrifuged briefly, resuspended in lysis buffer (250 mM KAc, 10 mM MgAc_2_, 50 mM HEPES, pH 7.4, 0.1% NP40) and homogenized in the presence of a protease inhibitor cocktail. The lysate was clarified by centrifugation at 26,000 *g* for 1 h and the supernatant was collected [45]. Approximately 100 µl of a streptavidin-agarose bead suspension (Molecular probes, S-951) pre-washed in binding buffer (lysis buffer without NP40) was added to the supernatant and incubated on a rotary shaker at room temperature for 1 h. The beads were then washed several times by centrifugation to remove unbound proteins followed by addition of SDS-PAGE lysis buffer and boiling for 3 min. The lysate was fractionated by electrophoresis under reducing conditions on a 10% acrylamide-SDS gel and stained with Coomassie Blue. The spots corresponding to proteins that reacted with the SM1 antibody were excised, washed and digested with trypsin prior to LC/MS/MS analysis as described below [46],[47].

### MS analysis

The digested sample was loaded online onto a fused silica capillary column (12 µm of YMC gel ODS-A). The peptides were separated with a linear gradient elution from 95% mobile phase A to 45% mobile phase B [90% acetonitrile with 10% H2O, 0.4% acetic acid, and 0.005% heptafluorobutyric acid (vol/vol)] in 40 min. A potential of 2.7 kV was applied to the emitter in the ion source. The mass spectra were acquired on a Micromass-Waters (Manchester, U.K.) quadrupole time-of-flight (Q-TOF) API-US mass spectrometer equipped with a nanoelectrospray ion source (Proxeon Biosystems, Odense, Denmark). The acquisition and the deconvolution of data were performed on a MassLynx Windows NT PC data system (version 4). All spectra were obtained in the positive-ion mode. The processed tandem MS spectra were searched against the NCBI non-redundant protein database. Searches were performed with Mascot version 1.9 [48].

### Blocking sporozoite invasion with anti-saglin antibody

Starting 10 days after feeding *An. gambiae* mosquitoes on a *P. berghei*-infected mouse, 5 to 10 mosquitoes were dissected daily and checked for sporozoite invasion of salivary glands. As soon as the first infected salivary gland was detected, sometime between day 14 and day 16, 1.5 to 2.0 µg (total ascites protein) of anti-saglin monoclonal 2A3 antibody supernatant [7] or the same amount of unrelated control antibody (normal mouse ascites) in *Aedes* saline [49] was injected into the hemocoel. At 48 h after injections, salivary glands were dissected and transferred to a tube containing 100 µl of PBS, and homogenized to liberate the sporozoites. These were collected by centrifugation at 6,000 RPM for 5 min, the pellet was resuspended in 20 µl of PBS, and sporozoites were counted using a hemocytometer.

### Expression and purification of recombinant TRAP domain-A and saglin proteins

The TRAP domain-A and the TSR-plus domain were expressed as GST fusion proteins and purified according to Jethwaney et al. [27]. Full-length saglin (GenBank accession # AAW31598) was cloned, expressed and purified according to Guzman et al. [50]. Briefly, a 1.2 kb fragment of Saglin was amplified using the forward (5′-CACCATGTCACGTTTGCCCACTGTACTGTTGCTC-3′) and reverse (5′-AAAGCCGGACGACTTGCGCGACTCGCTCAGATA-3′) primers and cloned into the pBAD expression vector (Invitrogen). Induction was done with arabinose and the expressed protein (His-tagged at the C-terminus) was purified using a nickel column.

### Site-directed mutagenesis

The Thr_126_-to-Ala TRAP domain-A point mutation was generated with the QuickChange Site-Directed mutagenesis kit (Stratagene). Briefly supercoiled plasmid DNA (10 ng) was annealed with two complementary primers containing the mutation Threonine 126 to Alanine (T_126_ to A ) : 5′-CACCACTTGGTACTGCAAATTTAACGGAGTGC-3′ and 5′-GCACTCGTTAAATTTGCAGTACCATGTGGTG-3′, and amplified for up to 15 cycles. The amplified product was digested with Dpn1 and transformed in XL1Blue supercompetent cells. The mutant protein was expressed in BL21(DE3) host cells and purified as described by Jethwaney et al [27].

### Immunofluorescence assays

Fixed salivary glands were incubated with wild type and mutant TRAP domain A, (2.5 µg/ml), washed, blocked and incubated with anti-TRAP domain-A antibody. Detection was done with anti-rabbit IgG conjugated with Alexa Flour-488.

Histidine-tagged saglin (2 µg/ml) was immobilized on Ni-NTA agarose beads, washed and allowed to bind to the purified wild type and the mutant TRAP domain A recombinant protein (2.5 µg/ml) in binding buffer (25 mM, Tris-HCl, 0.15 mM NaCl, and 8.0 mM MnCl_2_, pH 7.2) for 1 h at room temperature. Beads were washed and incubated with anti-domain-A antibody. Detection was done after incubation with Alexa Flour-568-conjugated anti-rabbit IgG

### RNA interference

A 400 bp fragment from the 5′ region of the saglin gene was amplified using a modified primer pair that incorporates the T7 primer sequences at their 5′ ends (forward: T7+ 5′-AGCGTTAGTGCCGAGACGATGAAA-3′; reverse: T7+ 5′-TTCGTTTCGCCGATCGCAATGTTC-3′). Another 530 bp fragment close to the 3′ end of the saglin coding region was also amplified using T7-modified primers (forward: T7+ 5′-AACATTGCGATCGGCGAAACGAAG-3′; reverse: T7+ 5′-GGCCGATAAACTGCTCGCAAATGT-3′). PCR products were cleaned with chroma spin columns (Stratagene, Texas) and double-stranded RNA (dsRNA) was synthesized and purified with the Ambion Mega-script kit (Ambion, Texas) according to the manufacturer's protocol. GFP control dsRNA was synthesized and purified as previously described [51]. Initial experiments using 200 or 400 ng of double stranded saglin RNA proved ineffective in down-regulating saglin expression. However, with 2.5 µg of dsRNA per mosquito [52] down-regulation was effective. The dsRNAs were dissolved in *Aedes* saline (150 mM NaCl, 4 mM KCl, 3 mM CaCl_2_, 1.8 mM NaHCO_3_, 0.6 mM MgCl_2,_ and 25 mM HEPES pH 7.0) [49]. Mosquito mortality due to injection of saglin dsRNA and GFP dsRNA was similar.

Starting on day 7, the salivary glands of 5 *P. falciparum*-infected *An. gambiae* mosquitoes were dissected daily and checked for the presence of sporozoites. About 2–2.5 µg of saglin or GFP dsRNA was injected into the hemocoel on the day when sporozoite invasion of the salivary glands was first observed (usually between day 14 and 16). Salivary glands were dissected 6 days after dsRNA injection and sporozoites were counted using a hemacytometer.

## Supporting Information

Figure S1 Binding pattern of 8 different lectins. Fixed salivary glands were incubated with FITC-labeled lectins and binding was detected by fluorescent microscopy. For each gland, a fluorescent image is shown in the upper panel and a DIC image in the lower panel.
(4.59 MB TIF)

Figure S2 Effects of glycosidase and protease treatments of salivary glands on SM1 peptide binding. Treatments: (A) N-glycosidase (PNGPase), (B) O-linked glycosidase and (C) EndoH. Control (a,b) and treated (c,d & e) glands were incubated with a mixture of jacalin and SM1. DIC images of the control and treated glands are shown in b and e. Fluorescence due to jacalin binding is green and SM1 binding is red.
(4.57 MB TIF)

Figure S3 Identification of SAGLIN by LCMS/MS analysis. (A) Total Ion Chromatogram (TIC) obtained from an LC-MS/MS run of the peptides derived from in-gel trypsin digestion of a SDS-PAGE upper band (see Figure 3B, arrows). (B) Mass spectrum of a small section of the TIC shows two doubly-charged ions (indicated by arrows) corresponding to saglin peptides that elute approximately at 24 and 28 minutes. (C,D) Product ion MS/MS spectrum of the doubly charged ions at *m*/*z* 511.32 and *m*/*z* 602.85 corresponding to the peptide sequences QLYDDLVR and EDLPVYQANR respectively, that matched the saglin protein.
(0.40 MB TIF)

Figure S4 Schematic of the *Plasmodium* TRAP protein and alignment of domain-A amino acids with the SM1 peptide. Previous work [21] has determined that threonine (T) 126 and aspartate (D) 157 in the MIDAS region of domain-A are crucial for salivary gland invasion. SP: signal peptide; TSR: throbospondin type I repeat; TM: transmembrane domain; Pf: *P. falciparum*; Pg: *P. gallinaceum*; Pk: *P. knowlesi*; Pv: *P. vivax*; Py: *P. yoelii*; Pb: *P. berghei*. Adapted from [53].
(0.32 MB TIF)

Figure S5 Interaction between salivary gland membrane-bound saglin with recombinant TRAP domain-A. Wells from a 96-well plate were coated with anti-saglin monoclonal antibody (10 µg/ml ascites). Crude midgut or ovary membranes (prepared as in [54]; equal protein amount) were captured onto the coated wells and washed to remove excess membranes. Wells were blocked and incubated with recombinant TRAP domain-A protein (2.5 µg/ml) followed by incubation with rabbit anti-domain-A antibody (1:1000 dilution). The wells were then incubated with alkaline phosphatase-conjugated goat anti-rabbit secondary antibody (1:5000 dilution). After washing, the wells were incubated with a chromogenic alkaline phosphatase substrate to quantify the amount of secondary antibody bound. The graph shows results with the complete protocol with salivary gland (SG) membranes (first bar from the left), complete protocol with ovary (Ov) membranes (second bar), protocol with SG membranes but with wells not covered with anti-saglin antibody (third bar), protocol with SG membranes but omitting incubation with recombinant domain-A protein (fourth bar).
(0.11 MB TIF)
