# Supplementary figures and images for: Malaria Parasite Invasion of the Mosquito Salivary Gland Requires Interaction between the Plasmodium TRAP and the Anopheles Saglin Proteins
Source: PLoS Pathog. 2009 Jan 16;5(1):e1000265. doi: 10.1371/journal.ppat.1000265 (PMC2613030; doi:10.1371/journal.ppat.1000265)

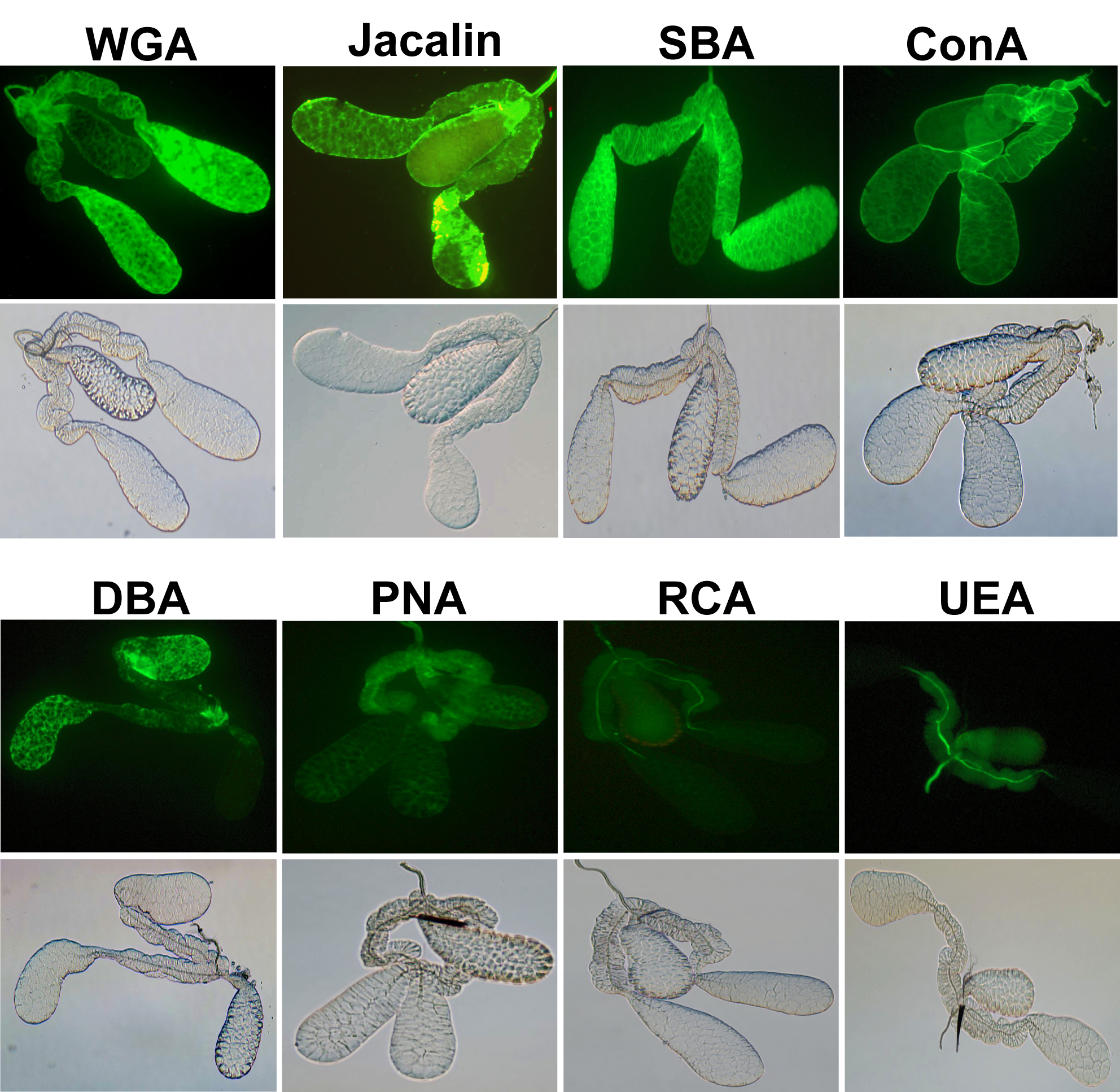

Supplement: Figure S1 — Binding pattern of 8 different lectins. Fixed salivary glands were incubated with FITC-labeled lectins and binding was detected by fluorescent microscopy. For each gland, a fluorescent image is shown in the upper panel and a DIC image in the lower panel. (4.59 MB TIF) [file ppat.1000265.s001.tif]

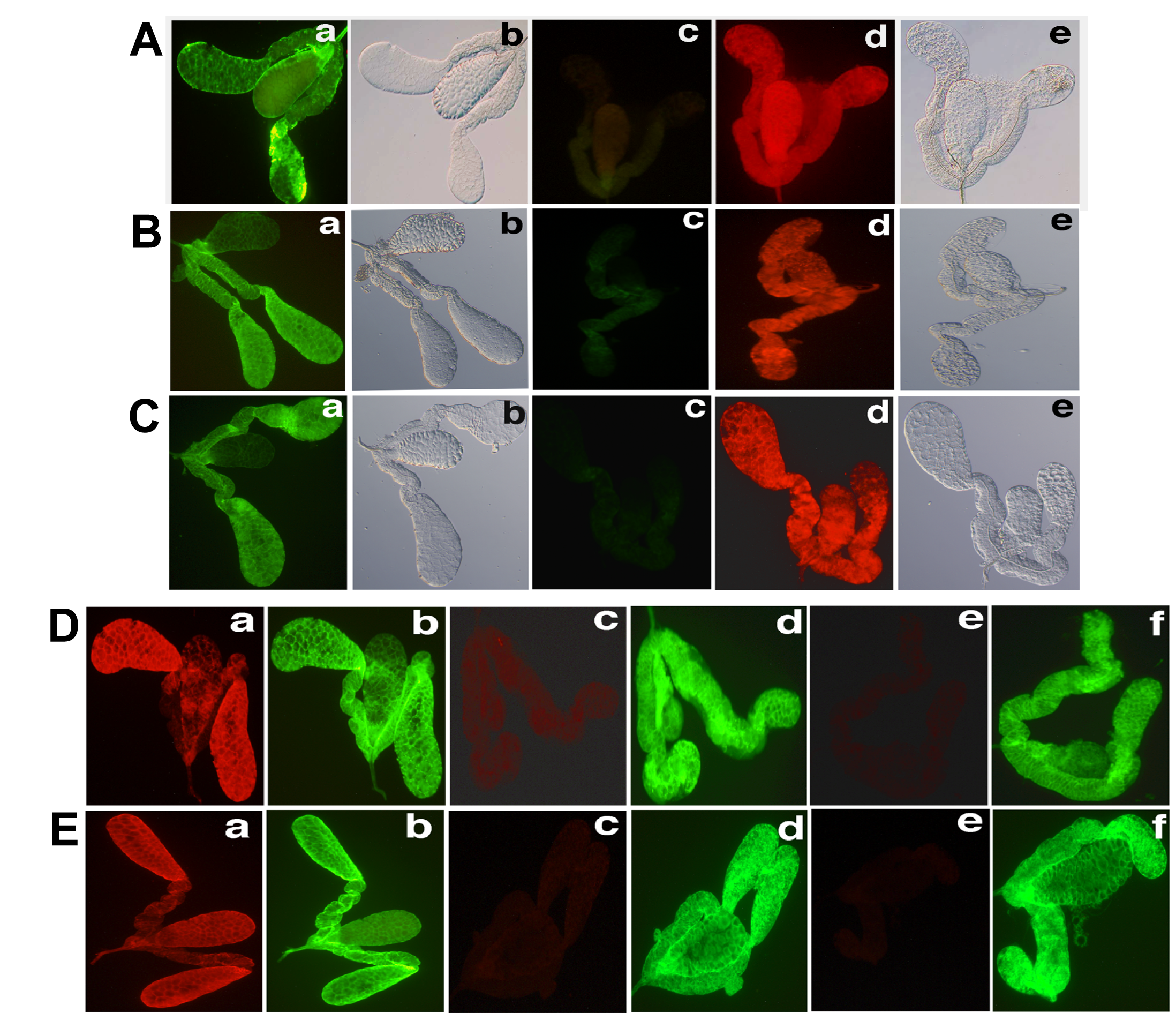

Supplement: Figure S2 — Effects of glycosidase and protease treatments of salivary glands on SM1 peptide binding. Treatments: (A) N-glycosidase (PNGPase), (B) O-linked glycosidase and (C) EndoH. Control (a,b) and treated (c,d & e) glands were incubated with a mixture of jacalin and SM1. DIC images of the control and treated glands are shown in b and e. Fluorescence due to jacalin binding is green and SM1 binding is red. (4.57 MB TIF) [file ppat.1000265.s002.tif]

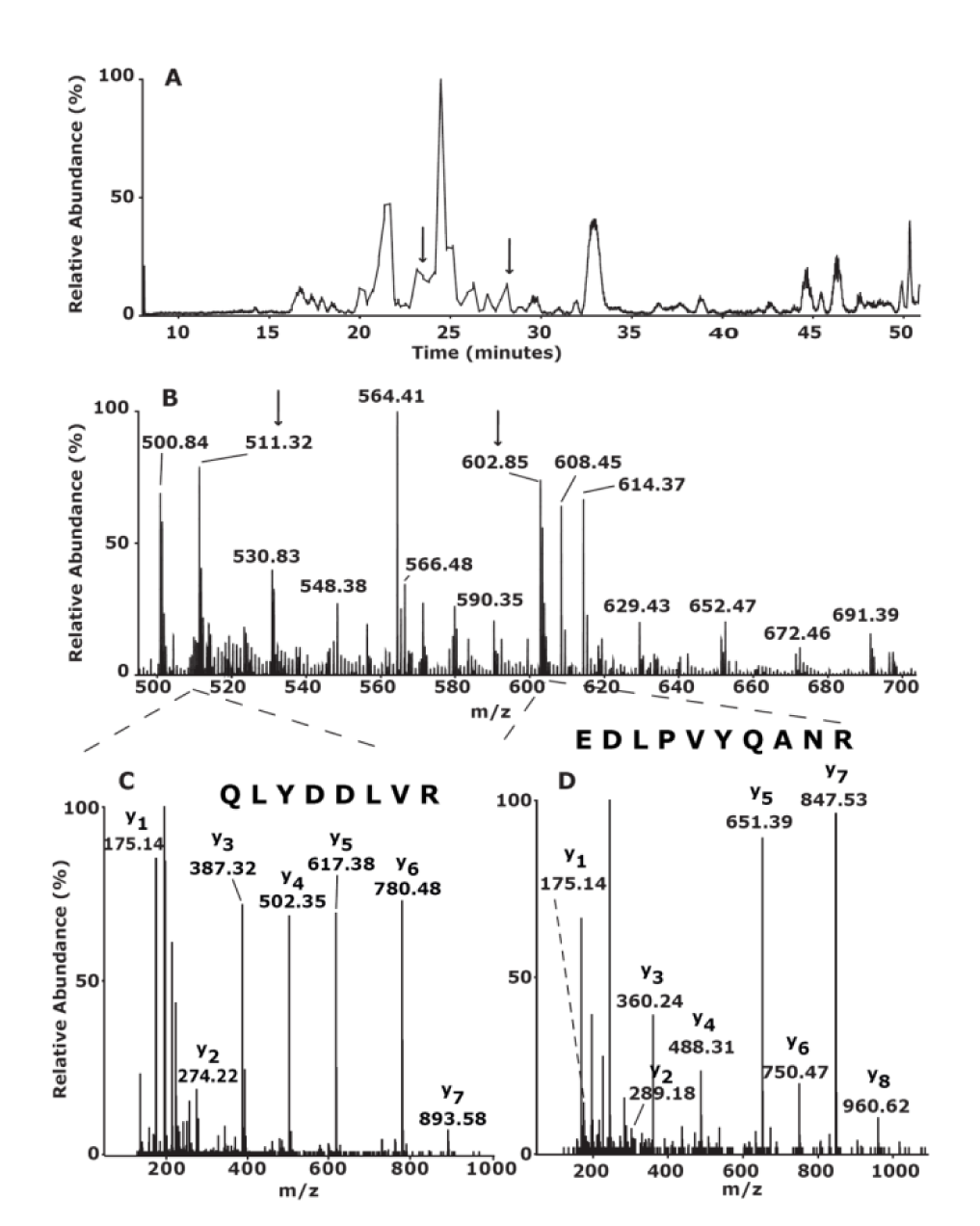

Supplement: Figure S3 — Identification of SAGLIN by LCMS/MS analysis. (A) Total Ion Chromatogram (TIC) obtained from an LC-MS/MS run of the peptides derived from in-gel trypsin digestion of a SDS-PAGE upper band (see Figure 3B, arrows). (B) Mass spectrum of a small section of the TIC shows two doubly-charged ions (indicated by arrows) corresponding to saglin peptides that elute approximately at 24 and 28 minutes. (C,D) Product ion MS/MS spectrum of the doubly charged ions at m/z 511.32 and m/z 602.85 corresponding to the peptide sequences QLYDDLVR and EDLPVYQANR respectively, that matched the saglin protein. (0.40 MB TIF) [file ppat.1000265.s003.tif]

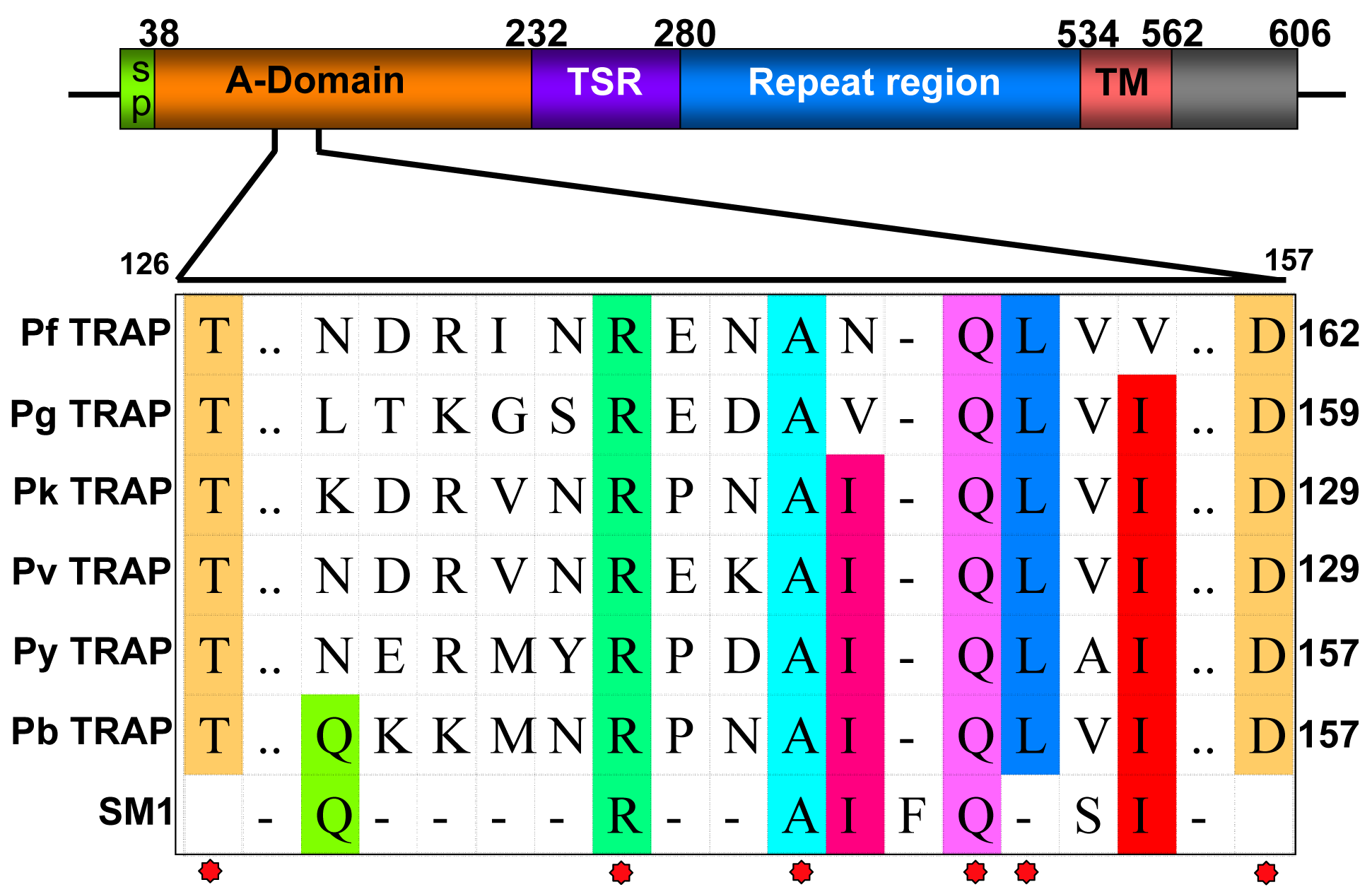

Supplement: Figure S4 — Schematic of the Plasmodium TRAP protein and alignment of domain-A amino acids with the SM1 peptide. Previous work [21] has determined that threonine (T) 126 and aspartate (D) 157 in the MIDAS region of domain-A are crucial for salivary gland invasion. SP: signal peptide; TSR: throbospondin type I repeat; TM: transmembrane domain; Pf: P. falciparum; Pg: P. gallinaceum; Pk: P. knowlesi; Pv: P. vivax; Py: P. yoelii; Pb: P. berghei. Adapted from [53]. (0.32 MB TIF) [file ppat.1000265.s004.tif]

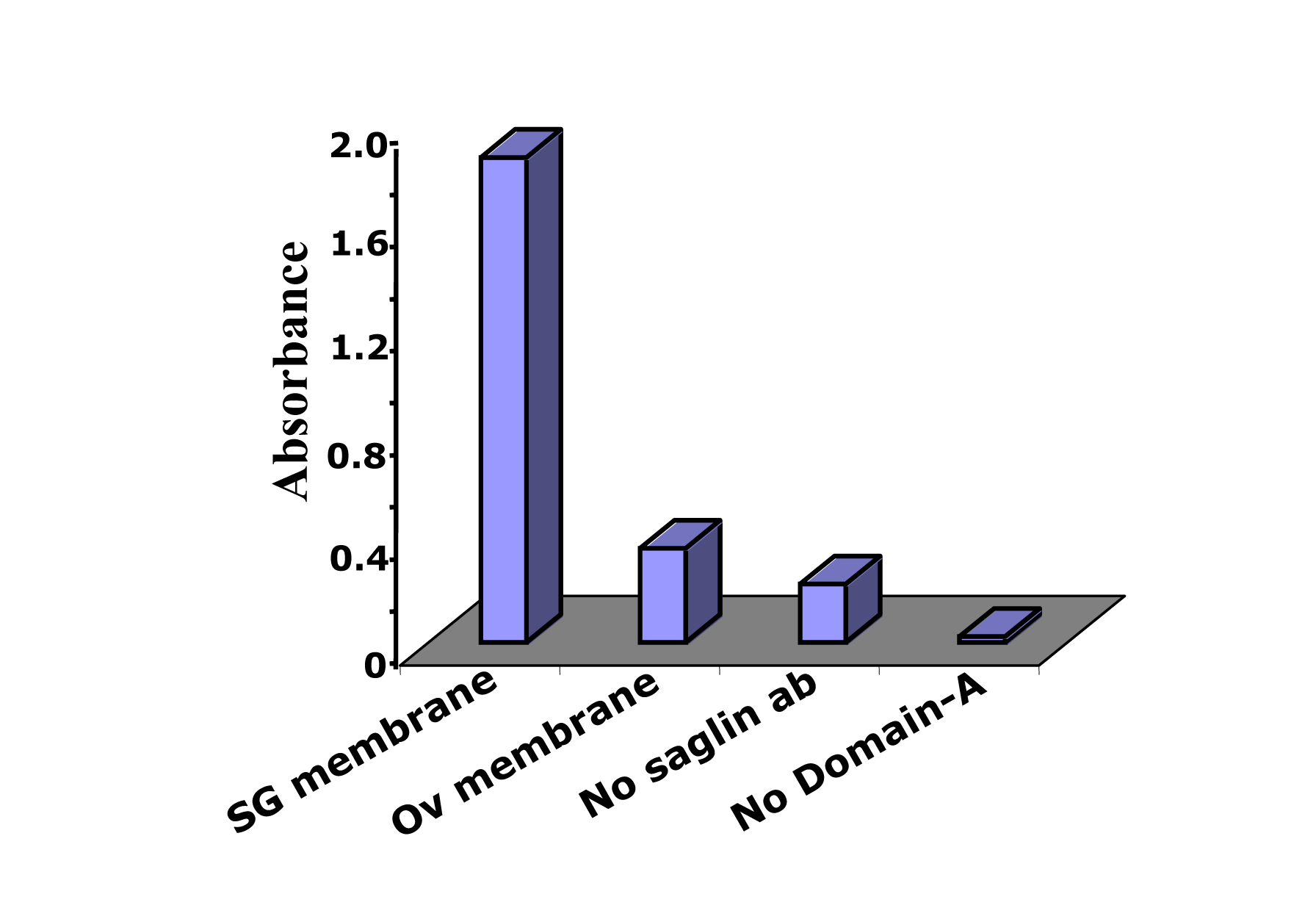

Supplement: Figure S5 — Interaction between salivary gland membrane-bound saglin with recombinant TRAP domain-A. Wells from a 96-well plate were coated with anti-saglin monoclonal antibody (10 µg/ml ascites). Crude midgut or ovary membranes (prepared as in [54]; equal protein amount) were captured onto the coated wells and washed to remove excess membranes. Wells were blocked and incubated with recombinant TRAP domain-A protein (2.5 µg/ml) followed by incubation with rabbit anti-domain-A antibody (1:1000 dilution). The wells were then incubated with alkaline phosphatase-conjugated goat anti-rabbit secondary antibody (1:5000 dilution). After washing, the wells were incubated with a chromogenic alkaline phosphatase substrate to quantify the amount of secondary antibody bound. The graph shows results with the complete protocol with salivary gland (SG) membranes (first bar from the left), complete protocol with ovary (Ov) membranes (second bar), protocol with SG membranes but with wells not covered with anti-saglin antibody (third bar), protocol with SG membranes but omitting incubation with recombinant domain-A protein (fourth bar). (0.11 MB TIF) [file ppat.1000265.s005.tif]
